# Enhancing the Efficiency of Membrane Processes for Water Treatment

**DOI:** 10.3390/membranes11030215

**Published:** 2021-03-19

**Authors:** Ibrahim M.A. ElSherbiny, Stefan Panglisch

**Affiliations:** 1 Chair for Mechanical Process Engineering and Water Technology, University of Duisburg-Essen, Lotharstr. 1, 47057 Duisburg, Germany; 2 DGMT German Society of Membrane Technology, Geschäftsstelle ZWU, Universitätsstr. 2, 45141 Essen, Germany; 3 IWW Water Center, Moritzstr. 26, 45476 Mülheim an der Ruhr, Germany

Pressure-driven membrane processes, i.e., microfiltration (MF), ultrafiltration (UF), nanofiltration (NF) and reverse-osmosis (RO), are globally recognized as an essential element in sustainable water management systems, thanks to their intrinsic advantages when compared to conventional processes, as well as to their versatility. Today, pressure-driven membrane processes are used for water treatment, purification, and reuse. Despite the successful development in recent decades of membrane filtration up to the industrial scale, huge interest in the optimization of membrane processes still exists. This has been realized so far through various approaches—for instance, the introduction of ultralow pressure or low fouling membranes, improving membrane retention and/or selectivity, increasing membrane life span, in addition to reducing energy consumption and the use of chemicals. In addition to advanced membrane materials and module design, the optimization of membrane processes or combining them with other treatment processes have increasingly become the focus of engineering researchers. The ultimate objective is commonly to further promote membrane process efficiency towards more sustainable, cost-effective, and environmental-friendly water treatment applications.

This Special Issue is devoted to “Enhancing the Efficiency of Membrane Processes for Water Treatment”. Several research articles are reporting promising results regarding boosting membrane-based separation processes in lab-scale experiments, pilot-scale (or case studies), and simulation studies. In the following, these articles will be briefly introduced.

Pressure-driven membrane filtration processes already have a long track-record in drinking water and wastewater treatment. Nevertheless, there are various aspects that require intensive research toward better understanding of the processes involved, further optimization of the technology, and increasing the applicability in other fields. For instance, the influences of operating pressure and groundwater pH on permselective properties of NF and RO membranes were studied in a pilot study by Cai et al. [1]. The feed water was a natural groundwater from the Eslalei region in northern Tanzania with high strontium and organic matter contents. Better strontium and organic matter removal by the NF membrane was measured by increasing the applied pressure and decreasing the groundwater pH to an acidic condition, because of increasing electrostatic repulsion at the membrane surface or modifications in organic matter interactions. Nevertheless, a pronounced decrease in strontium and organic matter retention was noticed for the NF membrane at the alkaline pH condition. Slightly enhanced strontium retention and weakened organic matter removal were found for RO membranes. Even though the NF membrane could not achieve adequate desalination (particularly for strontium), it was concluded to be suitable for investigating strontium–organic matter transport mechanisms using real groundwater.

Additionally, the re-adaptation or re-shaping of intrinsic membrane characteristics have been recently proposed to overcome the performance limitation of currently employed membranes in the market and enhance furtherly the membrane filtration processes. For instance, polyether sulfone Multibore^®^ ultrafiltration membranes were modified, in a study by Niestroj-Pahl et al. [2], by applying polyelectrolyte multilayers via the layer-by-layer (LbL) technique to promote their rejection capabilities towards salts and antibiotic resistance genes. The modification was successful in significantly reducing the barrier pore diameter from 20 nm to about 2 nm for eight double-layer (DLs) [3]; also, the molecular weight cut-off for 4.5 DL was measured to be 384 Da [2]. Moreover, the zeta potential measurement was specially adapted by Dillmann et al. to characterize the alteration of the membrane surface charge inside the capillaries upon alternating coating with polyelectrolyte multilayers [3]. The developed capillary NF membranes could completely retain DNA fragments 90 to 1500 nt (nucleotides) in length [2]. They also exhibited a typical NF performance, with 80% retention for MgSO_4_ and CaCl_2_ salts, and 23% retention for NaCl. Furthermore, the modified membranes were found to be stable toward mechanical backwashing (up to 80 L/(m²·h)) and chemical regeneration, in acidic and basic/oxidizing conditions.

In parallel, new polyether sulfone-based dopamine-modified graphene nanocomposite membranes were developed using a two-stage phase inversion method for ultra-low pressure RO applications [4]. In this method, dry phase inversion via solvent evaporation was carried out prior to the typical liquid-induced phase separation process in water. The characteristics of the developed membranes (e.g., contact angle, water uptake/swelling, porosity, and pore size distribution) were influenced by the interval of drying, or solvent evaporation, before precipitation in water. The new nanocomposite membranes exhibited superior salt retention (>99%), which was stable over an operating pressure range of 1–8 bar, as well as competent antifouling properties.

Enhancing the membrane filtration processes has also recently been realized via advanced operating procedures. Biofouling is still a major concern for numerous RO applications. UV pretreatment of the feed stream showed some promising results; however, the technology is not yet established because its effects are not sustainable. In a response, Sperle et al. have investigated the impact of in situ low fluence UV pretreatment, using UVC light-emitting diodes, on the performance of commercial low-pressure RO membrane in accelerated biofouling experiments, besides whether it might have a lasting effect on the biofilm [5]. It was revealed in this study that a low fluence of 2 mJ·cm^−2^ could delay the biofilm formation by more than 15% in lab-scale experiments. This was interpreted by the possible inactivation of cells in the feed stream, change in the adhesion properties and/or an induced cell cycle arrest. Moreover, the formed biofilms exhibited > 40% reduction in the hydraulic resistance, which might be caused by an altered microbial community, reduced adenosine triphosphate levels per cells, possibly impacting quorum sensing and extracellular polymeric substances production. Nevertheless, further research is still required to understand the mechanisms that govern the delayed and altered biofilm formation, as well as to investigate the transferability of the results to full-scale application, where multiple cleaning cycles and several membrane modules in series are applied.

Additionally, Venne et al. have explored the ability of ozone chemically enhanced backwashing to mitigate irreversible organic fouling by cyanobacteria microalgae, *Microcystis aeruginosa*, and improve the performance of ceramic UF membranes [6]. Batch ozonation tests showed that ozone reacts rather rapidly with feed water organic foulants rather than cyanobacteria cells. Nevertheless, during membrane filtration experiments, the combination of ozone treatment and hydraulic backwashing force was effective in reducing the fouling layer resistance by 35% more than in case of sole hydraulic backwashing. The ozone was postulated to weaken the compressible fouling layer formed by cyanobacteria, increase its porosity, and therefore, it became more hydraulically backwashable (i.e., reversible).

Furthermore, adaptation processes have been extended to include system design with respect to membrane module arrangement. The study by Barambu et al. has explored the ability of a tilted panel membrane filtration system to treat real laundry wastewater, aiming at water and detergent reuse [7]. The research results showed the potential of the new system in mitigating membrane fouling by promoting air bubbles’ contact with the membrane surface, and consequently, inducing continuous foulants detachment. The membrane permeability could be substantially enhanced by 83% by adjusting the aeration rate and membrane tilting angle, in comparison with an unaerated membrane set-up. Besides, a detergent recovery of 32% could be achieved. Accordingly, the proposed system is a potential approach for fouling management and detergent recovery.

Another potential approach for enhancing the efficiency of membrane separation processes for water treatment is based on the combination, or hybridization, with other well-established or new treatment technologies. Despite the fact that this approach could be promising in terms of the consolidation of versatile treatment processes advantages and the control of the permeate quality, the design, and/or operation, of such hybrid systems, as well as the costs and energy consumption are currently the major challenge.

The pressurized powdered activated carbon/coagulation/ceramic MF hybrid system (PAC/Alum/MF) was investigated at pilot scale for treating low turbidity and low natural organic matter surface water spiked with organic microcontaminants (e.g., pesticides, pharmaceutical compounds, microcystins) [8]. PAC/(Alum)/MF achieved a 75–97% removal of total microcontaminants, using 4–18 mg·L^−1^ of mesoporous PAC and contact time of 2 h, along with reliable particle separation (turbidity < 0.03 NTU) and low aluminum residuals. Microcontaminants showed different amenabilities to PAC adsorption, depending on their charge, hydrophobicity (Log K_ow_), polar surface area and aromatic rings count. PAC/Alum/MF attained natural organic matter removal of 29–47% and decreasing trihalomethane formation potential (THMFP) by 26%. The addition of PAC promoted natural organic matter removal by coagulation (+15–9%); however, no substantial improvement in THMFP and membrane fouling was noticed. Moreover, the PAC/Alum/MF system was found to be an effective barrier against oxidation-resistant microorganisms, i.e., aerobic endospores (as indicators for protozoan oocysts). PAC dosing was crucial for removing bacteriophages, indicators for viruses (≥1.1-log reduction).

In parallel, the removal of trace organic contaminants (TrOCs) from Rhine bank filtrates employing a semi-technical drinking water treatment plant comprising two parallel processes, low-pressure reverse osmosis (LPRO) and activated carbon filtration (ACF), was investigated at pilot-scale [9]. TrOCs comprised polyfluorinated compounds, pharmaceuticals, pesticides, metabolites, volatiles, nitrosamines and aminopolycarboxylic acids. Generally, a stable LPRO membrane performance along with limited membrane fouling was reported. Nevertheless, membrane autopsy studies revealed that the leading module suffered from biofouling, while scaling deposits of calcium, silicate, barium and strontium were seen in the last module. The rejection of TrOCs by LPRO was related to TrOCs’ molar masses and Log K_ow_ values; TrOCs with a molecular weight ≥ 150 Da were completely retained. However, volatile halogenated compounds were detected in the permeate due to the low rejection of dichloro and trichloro compounds by LPRO. In parallel, ACF showed a significant removal for almost all TrOCs, whereas salts were not removed. The removal of TrOCs was reported to be dependent on the specific throughput. Subsequently, the authors have proposed a parallel operation of ACF and LPRO as an optimal treatment solution in cases where both TrOCs and hardness are required to be reduced, depending on the water source condition.

Furthermore, the ability of an integrated electrocoagulation–NF membrane system for the treatment of textile wastewater was investigated [10]. Electrocoagulation experiments were performed using iron electrodes in an electrolytic cell integrated with a nanofiber nylon NF membrane for separating the flocculants. The integrated system achieved a dye removal efficiency of 79%, using 1000 ppm of NaCl as the electrolyte and 2 V (corresponding to energy input of 1.65 kWh·m^−3^), and model feed water comprising 10 ppm celestine blue dye at pH 7. This was essentially higher than the dye removal efficiency in the case of the standalone electrocoagulation process and/or membrane filtration. Nevertheless, the nanofiber membrane was reusable twice only, therefore further research toward nanofiber membranes with improved mechanical strength is required.

Besides the pressure-driven membrane processes, membrane distillation (MD) is becoming more popular in the last few years due to advancements in membrane material and process control. An example for process optimization in this context is the study by Blankert et al. [11]. The trade-off between the evaporation efficiency and driving force efficiency in an MD system was thermodynamically studied, by analyzing the heat flow in a single effect MD system. A non-zero net driving temperature difference was found as the minimum net driving temperature difference for a rational operational strategy. Below this limit, both productivity and exergy consumption can be improved by increasing the net driving temperature difference. This limit was revealed to depend on the properties of the membrane and the heat exchanger. Subsequently, the minimum net driving temperature difference concept can be used for membrane and process optimization.

Membrane processes are a recognized essential element in sustainable water management. Looking back, we can see that the first large membrane-based water treatment plants were commissioned as early as the 1960s. Since then, membrane technology has continuously evolved, always accompanied by excellent research, with the goals of understanding fundamental processes, reducing investment and operating costs, and opening up further areas of application. The articles published so far in this Special Issue are the best examples of this statement. As guest editors, we are especially grateful for all these contributions, and thank the authors, reviewers, and publisher for their outstanding work.
